# Scoring systems for early prediction of tibial fracture non-union: an update

**DOI:** 10.1007/s00264-021-05088-0

**Published:** 2021-06-15

**Authors:** George D. Chloros, Nikolaos K. Kanakaris, James S. H. Vun, Anthony Howard, Peter V. Giannoudis

**Affiliations:** 1 Academic Department of Trauma and Orthopaedics, School of Medicine, University of Leeds, Clarendon Wing, Floor D, Great George Street, Leeds General Infirmary, Leeds, LS1 3EX UK; 2 NIHR Leeds Biomedical Research Center, Chapel Allerton Hospital, Leeds, UK

**Keywords:** Non-union, Fracture, Scoring systems, Scores, LEG-NUI, FRACTING, TFHS, NURD

## Abstract

**Purpose:**

To evaluate the available tibial fracture non-union prediction scores and to analyse their strengths, weaknesses, and limitations.

**Methods:**

The first part consisted of a systematic method of locating the currently available clinico-radiological non-union prediction scores. The second part of the investigation consisted of comparing the validity of the non-union prediction scores in 15 patients with tibial shaft fractures randomly selected from a Level I trauma centre prospectively collected database who were treated with intramedullary nailing.

**Results:**

Four scoring systems identified: The Leeds-Genoa Non-Union Index (LEG-NUI), the Non-Union Determination Score (NURD), the FRACTING score, and the Tibial Fracture Healing Score (TFHS). Patients demographics: Non-union group: five male patients, mean age 36.4 years (18–50); Union group: ten patients (8 males) with mean age 39.8 years (20–66). The following score thresholds were used to calculate positive and negative predictive values for non-union: FRACTING score ≥ 7 at the immediate post-operative period, LEG-NUI score ≥ 5 within 12 weeks, NURD score ≥ 9 at the immediate post-operative period, and TFHS < 3 at 12 weeks. For the FRACTING, LEG-NUI and NURD scores, the positive predictive values for the development of non-union were 80, 100, 40% respectively, whereas the negative predictive values were 60, 90 and 90%. The TFHS could not be retrospectively calculated for robust accuracy.

**Conclusion:**

The LEG-NUI had the best combination of positive and negative predictive values for early identification of non-union. Based on this study, all currently available scores have inherent strengths and limitations. Several recommendations to improve future score designs are outlined herein to better tackle this devastating, and yet, unsolved problem.

## Introduction

Non-union of long bones is a relatively frequent and most devastating complication of trauma with an incidence ranging from 5 to 10% [1, 2]. Management is typically long lasting and associated with considerable healthcare costs, tremendous impact on the patient health-related quality of life including family and psychological repercussions, as well as significant tax payer consequences as those patients do not return to work very frequently [3, 4]. Although, there is no accepted universal definition of “non-union”, the U.S. Food and Drug Administration (FDA) defines it as a fracture that is at least nine months old and has not shown any signs of healing for three consecutive months [5]. However, these are inappropriately long intervals and it is of utmost importance to be able to predict early which fractures will advance into non-unions in order to intervene promptly and, optimally, within 12 weeks after initial fracture fixation to prevent the aforementioned severe and multifaceted consequences. In this regard, radiographic non-union prediction scoring systems show some potential; however, there are issues with inter- and intra-observer reliabilities and their role is to supplement clinical judgement and laboratory data on a case-by-case basis [2]. Establishing biomarkers as prediction tools has not yet been effective or standardized [6, 7]. Recently, efforts have been made to develop clinical scoring systems [5, 8–10] to aid in the early prediction of non-union. The purpose of this study is therefore to provide an evaluation of the currently available clinical non-union prediction scores to the clinical setting of tibia fracture patients, analyze their strengths, weaknesses, and limitations.

## Materials and methods

In the *first part* of the study, a systematic search for the currently available non-union prediction scores was undertaken. All queries were performed in January 2021 by one reviewer. The databases queried included PubMed (1980–2020), MEDLINE (1980–2014), and EMBASE (1974–2020). The search strategy was as follows: (((non-union*) OR (non-union*)) OR (bone healing)) AND (predict*) and subsequently (((non-union*) AND (non-union*))) AND (scor*). The search included English, French and German languages. Inclusion criteria were studies that reported non-union scoring systems to predict non-union of long bone fractures. Exclusion criteria were: Papers that exclusively report radiographic non-union prediction scores, studies that evaluate and score established non-unions, non-fracture studies, non-long bones, animal studies, basic science articles, editorials, personal correspondence, conference proceedings and review articles. Furthermore, all the references from the included studies were scrutinized to ensure that no eligible studies are missing from the review.

The *second part* of the study consisted of comparing the validity of the non-union prediction scores found in the first part of the study. The database of our Level I trauma centre Institution, which consists of prospective data collection, was interrogated for patients who underwent intramedullary nailing of the tibia. In a consecutive manner, the authors randomly selected five of those patients who had non-union of their tibias and ten who developed union. To allow comparisons across the different scoring systems, the tibia, as well as only one method of treatment, i.e. intramedullary nailing, were investigated because they were the only common denominators in all systems. Clinical and radiological data for each patient was evaluated firstly by two observers (***blinded***), and the results were confirmed by two senior Major Trauma Center consultants (***blinded***).

## Results

### Available non-union prediction scoring systems

The literature review search revealed the following four clinical non-union prediction scoring systems available: The Leeds-Genoa Non-Union Index (LEG-NUI) [5], the Non-Union Determination Score (NURD) [10], the FRACTING score [9], and the Tibial Fracture Healing Score (TFHS) [8]. The scores are briefly summarized in Table 1.

**Table 1:** Non-union prediction scoring systems. (*^1^): Parameters pertaining to plate fixation are not mentioned. (*^2^): Post-fixation cortical contact of 0% is excluded in the NURD score, as these fractures are considered “a priori” at high-risk for nonunion. IMN: Intramedullary Nailing

Non-union prediction scoring system (applied to Tibia fractures)		Summary of parameters examined				Score range (min–max)	Segment of bone involved	Method of treatment	Thresholds reported	Comments
1	FRACTure HealING (FRACTING) Score [9]	12 Clinical parameters(*^1^)		5 Radiological parameters		2.5–25	41-A, 42-A-B and C, and 43-A and B (plateau excluded)	Surgeon preference (includes all possible modes of treatment)	Score ≥8 at immediate post-operative period	63% sensitivity, 81% specificity, and 53% positive predictive value
		1	Age	1	Stability					
		2	Malnutrition	2	Misalignment					
		3	Diabetes	3	Loss of bone substance					
		4	Smoking	4	Fracture of tibia alone					
		5	NSAID use	5	Bone diastasis					
		6	Fracture Exposure							
		7	Fracture location on bone							
		8	Implant device used							
		9	Bone graft requirement							
		10	Length of surgery							
		11	Preop hemoglobin							
		12	Postop hemoglobin							
2	LEeds-Genoa Non-Union Index (LEG-NUI) [5]	4 Clinical parameters		4 Radiological parameters		1 - 8	42A, B or C (tibial shaft only)	IMN, plate and circular frame, uniaxial external fixator excluded	Score ≥5 within 12 weeks	Scores of 1-4 and 5-8 are highly predictive of union and nonunion respectively (91% sensitivity and 77% specificity)
		1	Bone involved (tibia vs femur)	1	Type of fracture (complexity)					
		2	Extent of soft-tissue damage	2	Initial displacement					
		3	Method of reduction	3	Post-surgical fracture gap					
		4	Presence of Infection	4	Mechanical stability of fixation					
3	Non-Union Risk Determination (NURD) Score [10]	6 Clinical parameters		3 Radiological parameters		0 -21	Tibial shaft	IMN	Score ≥9 at immediate post-operative period	0-5: 2% of nonunion 6-8: 22% of nonunion 9-11: 43% of nonunion >12: 61% of nonunion
		1	Fracture Exposure	1	Low-energy fracture					
		2	Compartment syndrome	2	Spiral Fracture					
		3	Flap coverage requirement	3	% of cortical contact (*^2^)					
		4	Gender							
		5	ASA grade							
		6	Chronic disease							
4	Tibial Fracture Healing Score (TFHS) [8]	Clinical parameters – 3		Radiological parameter - 1		1 - 6	41-A, 42-A,B or C and 43-A	IMN	Score <3 at 12 weeks	96% sensitivity and 90% specificity in predicting the need for additional surgery secondary to nonunion
		1	Pain	1	adjusted Radiographic Union Scale in Tibial fractures[12] – (aRUST)					
		2	Function (eg. ability to weight-bear)							
		3	Pain on manipulation							

#### FRACTING score [9]

The FRACTING score was prospectively developed by a collaboration of 41 trauma centres throughout Italy in 2018 [9], and it has been designed to predict tibial fracture healing time when applied in the immediate post-operative period. It is not specific to one mode of treatment and applies to AO types 41-A, 42-A-B and C, and 43-A and B fractures. The end-point of fracture healing was solely clinical (“full weight-bearing without pain”), without radiographic reporting and follow-up was 12 months. Out of the 363 patients, 319 (88%) healed within 12 months and 44 (12%) had failure of healing, i.e. they had not healed or required unforeseen secondary surgical procedures. Score calculation relies on 12 clinical and five radiological parameters (see Table 1). Although the score calculation relies on radiographic parameters, no final radiographic evaluation is performed as far as union in the validation of this score. The minimum potential score is 2.5 and the maximum is 25. For the fractures that healed, the authors separated healing that took place after six months, versus healing that was complete within six months. Twelve percent of fractures with a score of ≤ 7, whereas 43% of fractures with a score of > 7 took more than six months to heal. In their discussion, the authors recommend a score of 8 with a sensitivity of 63%, specificity of 81% and 53% positive predictive value for fractures destined to heal in more than six months (associated with delayed union and non-union).

#### LEG-NUI score [5]

The LEG-NUI score was developed as a clinical decision rule from a retrospective case–control study of 100 patients with non-union versus 100 controls for either femur or tibia fractures that was performed in two level-I trauma centres, Leeds, UK and Genoa, Italy [5]. The end-point for non-union is the FDA definition, i.e. a fracture that is at least nine months old and has not shown any signs of healing for three consecutive months [5]. Modes of treatment evaluated included IMN, plate and circular frame, whereas patients with uniaxial external fixation were excluded. This score only applies to the shaft of the tibia, i.e. AO type 42-A, B or C. Furthermore, to eliminate confounders, significant bone gaps or segmental fractures were excluded. Initially, 10 risk factors for non-union were identified based on their prevalence in the literature; however, smoking and vascularization area of the diaphysis (upper, middle and lower diaphyseal thirds) were not taken into account in the final development of the score because they were found to be non-significant (p > 0.05). Therefore, a scoring system of a total of eight parameters (4 clinical and 4 radiological) to predict non-union within 12 weeks of treatment for either femoral or tibial shaft fractures was proposed (with a minimum score of 1 and a maximum score of 8) (see Table 1). Via a Receiver Operating Characteristics (ROC) curve an optimal cut-off was determined showing a 91% sensitivity, 77% specificity, with scores of 1–4 predictive of union and 5–8 highly predictive of non-union [5], and therefore, a threshold of ≥ 5 was recommended by the authors for the prediction of non-union.

#### NURD score

The NURD score was developed in 2016 by a group at the University of Maryland to predict tibial non-unions at the immediate post-operative period [10]. Only tibial shaft fractures specifically treated by reamed intramedullary nailing were studied and their non-union definition was those fractures that underwent unplanned secondary procedures for non-union [11]. At its outset, the score excludes patients with less than 3 mm cortical contact on post-operative radiographs (defined as “0%” cortical contact), to make sure that only patients who have at least some cortical continuity and therefore are expected to unite are included. Of their 382 patients, 326 went into union and 56 into non-union, requiring additional surgical procedures. To calculate the score, six clinical parameters and three radiological parameters are taken into account while the score ranges from 0 to 21 (see Table 1). NURD scores of 0–5, 6–8, 9–11 and > 12 have 2, 22, 43 and 61% chances of non-union, respectively [10]. Therefore, a NURD score of ≥ 9 has an ~ 50% chance of developing a non-union.

#### TFHS

The TFHS was recently developed as a simple, office-based tool to assess non-union [8]. AO fracture types 41-A, 42-A,B or C and 43-A treated with reamed intramedullary nail were studied, and their non-union definition was persistence of pain and lack of radiographic union for more than six months following the IMN procedure. Of the 87 patients studied, 77 healed whereas ten went into non-union. The score is calculated by adding three clinical and one radiological parameter, the “adjusted” RUST, which is basically the RUST score [12] divided by 4 (Table 1) and ranges from 1 to 6. Their results show that a score < 3 has a 96% sensitivity and 90% specificity in predicting the need for additional surgery secondary to non-union with positive and negative predictive values of 75 and 99% respectively.

### Clinical application of the scoring systems in 15 patients with tibial shaft fractures treated with intramedullary nailing

**Fig. 1:**
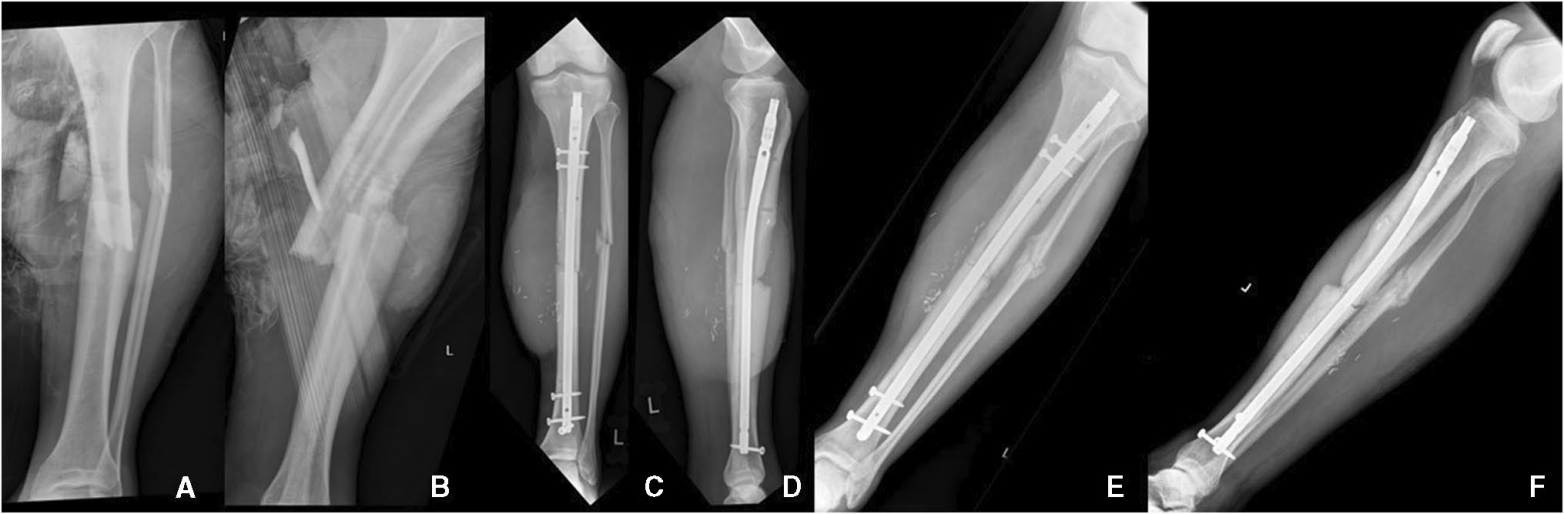
Non-union Group: Patient #1 (45 year-old male, sustained a IIIB fracture following a road traffic accident that required reconstruction with a gracilis free-flap). (**A**) AP and (**B**) Lateral radiographs on admission, (**C**) AP and (**D**) Lateral radiographs at the immediate post-operative period, (**E**) AP and (**F**) Lateral radiographs at the 3-month mark. FRACTING Score = 8, LEG-NUI = 5, NURD = 10. * For parameters used to calculate the score derived, refer to Table 1

**Fig. 2:**
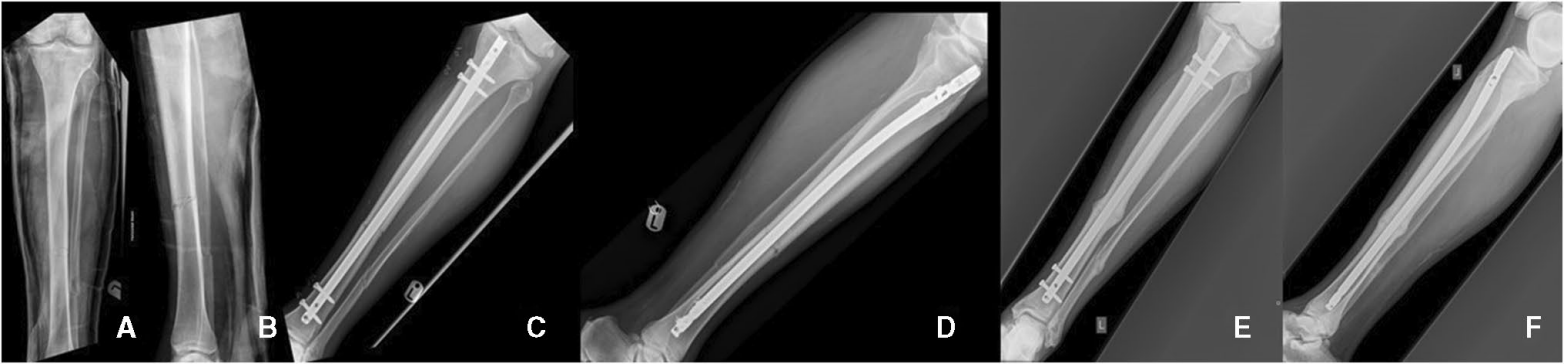
Union Group: Patient #6 (61 year-old male, sustained a grade II open fracture following a road traffic accident). (**A**) AP and (**B**) Lateral radiographs on admission, (**C**) AP and (**D**) Lateral radiographs at the immediate post-operative period, (**E**) AP and (**F**) Lateral radiographs at the 3-month mark. FRACTING Score = 7, LEG-NUI = 3, NURD = 5. * For parameters used to calculate the score derived, refer to Table 1

In the Non-Union group, there were five male patients, with mean age 36.4 years (range 18–50 years), whereas in the Union group there were ten patients (8 males, 2 females) with mean age 39.8 (range 20–66 years). The following score thresholds were used to calculate positive and negative predictive values for non-unions: FRACTING score ≥ 8 at the immediate post-operative period, LEG-NUI score ≥ 5 within 12 weeks, NURD score ≥ 9 at the immediate post-operative period, and TFHS < 3 at 12 weeks. Figures 1 and 2 show examples of patients from the Non-union and Union group respectively.

In the non-union group of patients, the FRACTING score threshold of ≥ 8 had an 60% positive predictive value (i.e., predicted that 3/5 would be problematic), see Table 2. In the union group of patients, the FRACTING score threshold of < 8 was present in 6/10 patients, and therefore had an 80% negative predictive value.

**Table 2:** Results of the Clinical Application of the 3 out of 4 Non-Union Prediction Scoring systems. The TFHS was not included as there were insufficient data to retrospectively calculate it

	Patient #	FRACTure HealING (FRACTING) Score [9]	LEeds-Genoa Non-Union Index (LEG-NUI) [5]	Non-Union Risk Determination (NURD) Score [10]
NON-UNION Patient group (5)	1	8	5	10
	2	10	7	6
	3	5	5	4
	4	7	6	5
	5	10	6	11
Positive Predictive Value		60%	100%	40%
UNION Patient group (10)	1	7	5	4
	2	5	1	1
	3	2	1	2
	4	3	2	1
	5	4	3	0
	6	7	3	5
	7	5	2	5
	8	10	3	3
	9	5	2	2
	10	10	4	4
Negative Predictive Value		80%	90%	100%

The LEG-NUI score threshold of ≥ 5 in the non-union group of patients, had an 100% positive predictive value (i.e., predicted that 5/5 would be problematic). In the union group of patients, the LEG-NUI score threshold of < 5 was present in 9/10 patients, and therefore has a 90% negative predictive value (Table 2).

The NURD score threshold of ≥ 9 in the non-union group of patients, had an 40% positive predictive value (i.e., predicted that 2/5 would be problematic). In the union group of patients, all patients had a score of less than 9, and therefore a 100% negative predictive value (Table 2).

Regarding the TFHS score at three months, no patient had all the parameters of the score available for calculation and therefore it is not mentioned in Table 2. Specifically, “pain on manipulation” vs “no pain on manipulation” was not recorded for any patient in any of the routine clinic visit notes. Also, for three patients, there were no accurate recordings of pain status. Consequently, due to the parameter included that relates to ‘manipulation and generation of pain’ that was not available to evaluate, accurate computation of this score was not feasible.

## Discussion

Radiographical [2] and clinical non-union prediction scoring systems mentioned herein involve the tibia, as it has the highest incidence of fractures of all long bones resulting from trauma [13]. Furthermore, tibia fracture healing times are widely variable and may range from a minimum of six weeks to several months, sometimes requiring additional procedures causing significant disability and substantial direct and indirect costs [3, 4]. Currently, several endeavors, have been underway to help predict which fractures are at high-risk of non-union, by identifying potential factors [14], and some of them have been grouped together to develop the scoring systems analysed here. The score that showed the strongest prediction for non-union in this cohort of patients was the LEG-NUI with a 100% positive and 90% negative predictive values for non-union. In the LEG-NUI, it was shown that out of the 200 patients, 46 out of 47 patients with non-union would have benefited from prompt intervention by applying the LEG-NUI [5]. In addition, the strengths of this system are: (1) It is simple and easy to apply: the eight variables are clearly defined and practical as there is a binary system, i.e. yes or no answer to score each one; (2) It accounts for infection [15] and gives sufficient time for it, i.e. it allows calculation within the first 12 weeks. However, surprisingly smoking was eliminated as a factor after it was shown that it had no significant effect in the fracture healing. Although traditional evidence is that tobacco smoking is associated in delayed/non-union development [14], there have been also reports that dismiss it [16]. In the development of the NURD score, smoking was also found to be non-statistically significant and it was therefore eliminated as well [10]. Of note, the LEG-NUI system does not take NSAID use into account, as this particular cohort of patients discontinue their use, per institutional protocols. There is an app available on the itunes store (see Table 3) but no android or web-based version yet.

**Table 3:** Scoring systems for non-union prediction: strengths and weaknesses/limitations

Non-union prediction scoring system applied in tibia fractures		Strengths		Weaknesses/Limitations	
1	FRACTure HealING (FRACTING) Score [9]			1	Absence of standardized treatment for tibial fractures
				2	Absence of any radiographic parameter to assess healing
				3	Absence of clear definitions/guidance regarding several parameters to calculate the score
				4	Does not have a threshold to exclude “a priori” large gaps
				5	Evaluates at time zero, therefore not taking into account important variables e.g. infection.
				6	Lack of follow-up beyond 12 months from surgery. Ambiguity in the fractures healed after 12 months or undergoing secondary procedures: Not specified whether those were non-unions.
				7	363 patients treated by 79 authors of the paper (~4.6 patients per author): Large heterogeneity and therefore increased subjective bias in the data collection, treatment and outcomes.
				8	No currently available itunes, android or web-based version.
2	LEeds-Genoa Non-Union Index (LEG-NUI) [5]	1	Simple and easy to apply, i.e. binary system for each variable.	1	Does not account for NSAID use.
		2	Specific to the diaphysis: It is only valid for diaphyseal fractures which are known to be the most problematic for healing.	2	Not specific to the tibia: Study population consisted of both femur and tibia fractures.
		3	Excludes segmental tibial fractures that are known to be prone to delayed/non-union.	3	Currently available only on the itunes store (https://apps.apple.com/gb/app/leg-nui/id1504208110); no android or web-based version.
3	Non-Union Risk Determination (NURD) Score [10]	1	It can only be applied to patients who are expected to go into union (Excludes postoperative gaps of more than 3mm which may be a confounding factor for nonunion).	1	Specific to intramedullary nailing – no other methods of fixation studied
		2	Simple and easy to apply. Guidelines are specific.	2	90% were high-energy injuries with an average injury severity score of 16.5, therefore it does not cover the whole spectrum of tibia injury (i.e. low injury)
				3	Evaluates at time zero, therefore not taking into account important variables e.g. infections.
				4	No clear threshold for nonunion prediction is reported.
				5	Failed to predict nonunion when externally validated in a large cohort (SPRINT trial) [19].
				6	Currently available only as a web version (http://www.shocknurd.org/) with no android or itunes version.
				7	Failed External Validation [20]
4	Tibial Fracture Healing Score (TFHS) [8]			1	“Pain on manipulation” not a reliable indicator [21, 22].
				2	Limited applicability in retrospective studies.
				3	Currently not available as an smartphone or web-based app
				4	Specific to intramedullary nailing – no other methods of fixation studied

The FRACTING score showed only a moderate capability with a 60% positive and 80% negative predictive values for non-union prediction. In the FRACTING score, patients were followed up only until 12 months from the initial surgery and the end point to determine union was solely based on clinical grounds. It is stated that, “44 out of the 363 fractures healed after 12 months or underwent a second surgery” [9], but it is not specified which of these were “non-unions”, or why secondary procedures were required, which could be for a variety of causes. The FRACTING Score has the following important weaknesses:

1. Treatment of tibia fractures was not standardized as it was left to the surgeon’s discretion. This is important as it weakens the study results as fractures treated with different methods behave differently, as for example a tibia fracture definitively treated with a circular versus an axial external fixator.
2. There has been no centralized review of radiographs, no utilization of any radiographic scoring to assess healing, and the endpoint did not involve any radiological evaluation, and relied solely on clinical criteria. Although in some of the fractures the presence of callus was informally reported, it was not recorded in the results, and not taken into account at all. For every fracture the presence or absence of union should also be radiographically confirmed at a minimum: First of all, pain is subjective [17], may not be present in all patients, (e.g. Diabetics), and sometimes in clinical practice, a fracture treated with an IMN may still have a gap, and although not painful at the time, it becomes painful after the nail breaks secondary to a non-union.
3. Further weaknesses of the study include the vague and/or lack of definition and clear guidance for calculation in a standard manner of several of their parameters in their paper: Malnutrition, “unstable”, “loss of bone substance”, and “bone diastasis of > 2 mm”. This may create confusion to clinicians and may have affected the validity of the authors’ results.
4. It does not “a priori” exclude large gaps that are known to lead to non-union or delayed union.
5. It is calculated in the immediate post-operative period, and therefore variables such as infection that are known to impair healing and delay union [15] are not taken into account.
6. In their discussion section, the authors claim that the FRACTING score shows “good reliability” in assessing the “risk of non-union” with sensitivities, specificities and positive predictive values based on ROC curves, provided for a threshold of ≥ 8. However, immediately after that statement, the authors specify that application of this threshold helps to determine which fracture will “heal in more than six months”. As stated above, what happened to the 44/363 (12%) of fractures after 12 months, i.e. whether they healed or not, and which ones (and why) underwent a secondary procedure is not reported. In consequence, there is no way to know which of those were “non-unions”. Therefore, there is considerable ambiguity in their claims, and it seems like that this score has not specifically been addressed to predict non-union. Nevertheless, their stated threshold of ≥ 8 was used in our analysis.
7. A total of 363 patients have been treated by 79 authors of the paper, (i.e. ~ 4.6 patients treated per author): This introduces is a large amount of heterogeneity and therefore considerable subjective bias in the data collection, treatment and outcomes.
8. Τhere is no app, either mobile or web-based to help clinicians apply the score in a rapid and effective way. It may be concluded that this score has several inherent limitations that may limit its usefulness in the current clinical practice.

The NURD score aims at predicting non-union in cases where it is completely unexpected, therefore, it excludes patients who have 0% cortical contact, defined as > 3 mm on initial post-operative radiographs [10], and this was also done in our selection of patients. Therefore, to apply this score, we excluded patients with a > 3 mm post-operative gap. In addition, a threshold value of ≥ 9 was considered for the prediction of non-union, since patients with a NURD score of ≥ 9 have an ~ 50% chance of developing a non-union [10]. However, per the original paper, there is no established “universal” threshold for non-union prediction but rather a statement of probability of non-union based on score.

Like every other system that looks at non-union prediction scores at time zero, one of the weaknesses of the NURD is that it cannot account for infection. Therefore, by looking at patients from the same patient cohort that was used to establish the NURD [10], the same author group developed a non-union prediction model at six weeks post-operatively that included infection [18]. In the 323 patients studied, 50 (15%) went into non-union. The authors looked various variables predictive of non-union and determined that three variables at six weeks were predictive of non-union, including the NURD score (at time zero) [10], the presence of infection (deep infection requiring additional surgery) and the Radiographic Union Score for tibial Fractures (RUST) [12]. By looking at those three variables, they found an 82% sensitivity and 82% specificity for non-union [18]. They further subdivided their patients into three groups based on the RUST scoring into high (RUST ≥ 10), medium (RUST 6–9) and low (RUST < 6). In the first group, the NURD score made no difference, as all patients healed. A NURD score of ≥ 7 predicted non-union in 25% of patients with a medium RUST, and in 69% in the group with the low RUST (or those infected). Of note, even by looking at infection at six weeks, the authors excluded from their analysis 11 patients that had infection that was diagnosed after the six week point, i.e. almost 25% of the patients who had infection. Therefore, it seems reasonable to conclude that their six week point would be too premature for the evaluation of infection, whereas for that reason, in the LEG-NUI evaluated patients within the first 12 weeks [5]. In the patients studied herein, the NURD score showed the least positive predictive value for non-union, at 40%. This is confirmed by a recent paper, written in part by the same group of authors who originally developed the NURD score, 1276 patients from the “Study to Prospectively Evaluate Reamed Intramedullary Nails in Patients with Tibial Fractures” (SPRINT) trial [19] were used to externally validate the NURD score and it was found that the NURD score was unable to predict patients at high risk for non-union [20]. Although this was attributed to significant differences between the populations of the two studies, as for example the fact that 90% of the patients in the NURD study had high-energy injuries (average injury severity score of 16.5) [10], it clearly shows the limitations of the NURD score when applied to a more heterogenous group of patients, i.e. it lacks “generalizability”.

Finally, the authors of the TFHS state that it is considered to be a “simple office-based clinical tool” [8], in practice it may be hard to apply. First and foremost, pain “on manipulation” of a tibia fracture may not be a reliable parameter and it has been shown that manually assessing bone stiffness by orthopaedic surgeons is not reliable regardless of years of experience [21, 22]. Pain on palpation is also widely used by clinicians; however, there is a considerable variability in its subjective evaluation which depends on individual and cultural differences in pain perception and tolerance level [17]. Lastly, as it was shown in this study, those variables are not consistently recorded, which poses a serious limitation of the application of this score in retrospective studies.

Based on the above analysis and evaluation of the available non-union prediction scoring systems, some useful recommendations could be made:

1. Have a threshold fracture gap to exclude fractures that “a priori” are unlikely to heal and therefore strengthen the positive predictive value of the scoring system, as already done in the LEG-NUI [5] and NURD [10] scoring systems.
2. Scores should not be calculated at the immediate post-operative period: Sufficient time should be allowed for other parameters that may lead to the development of the non-union to be taken into account. For example, infection impairs healing and may be responsible for the development of a non- union [15], but this cannot be accurately assessed immediately after surgery.
3. Scores should be simple, and their calculation should be clear and practical:
  *Simple*: Each parameter should be specific, i.e. have a binary (yes/no) value to eliminate ambiguity.
  *Clear*: Very specific guidelines on how to complete each component should be readily available.
  *Practical/Cost-Effective*: Mobile phone and/or web-based applications should be made available in all platforms for efficiency for clinicians to be able to calculate them “on the fly”, on a “as needed basis” at the bedside. In the future, those may be linked automatically to the electronic medical records of the patients.
4. Scoring systems should be flexible enough to be applied in the clinical and research setting, including both prospective and retrospective studies.
5. Segmental tibia fractures should be accounted for separately, as they have significant complications and typically take longer to heal [16, 23]. Not excluded in the scores except LEG-NUI.

In summary according to the currently existing evidence, it appears that the LEG-NUI scoring system is associated with better accuracy and reliability. Prospective studies in the future would provide more evidence regarding the useability and predictability of the above developed scores to aid the clinical decision making for early intervention of this devastating quite common post-fracture fixation complication.

## Data Availability

The authors declare that the raw data are available.

## Code availability

N/A
